# Acrodermatitis Dysmetabolica as a Cutaneous Manifestation of Isoleucine Deficiency in Maple Syrup Urine Disease: A Systematic Review of Reported Cases

**DOI:** 10.1002/hsr2.73230

**Published:** 2026-09-22

**Authors:** Bahareh Abtahi‐naeini, Motahar Heidari‐Beni, Noushin Rostampour, Naeimeh Davoudi, Sarah Seyedyousefi

**Affiliations:** ^1^ Skin Diseases and Leishmaniasis Research Center Isfahan University of Medical Sciences Isfahan Isfahan Iran; ^2^ Pediatric Dermatology Division of Department of Pediatrics, Imam Hossein Children's Hospital Isfahan University of Medical Sciences Isfahan Isfahan Iran; ^3^ Child Growth and Development Research Center, Research Institute for Primordial Prevention of Non‐Communicable Disease Isfahan University of Medical Sciences Isfahan Isfahan Iran; ^4^ Metabolic Liver Disease Research Center Isfahan University of Medical Sciences Isfahan Isfahan Iran

**Keywords:** acrodermatitis, isoleucine, maple syrup urine disease

## Abstract

**Background and Aim:**

Maple Syrup Urine Disease (MSUD) is a metabolic disorder affecting branched‐chain amino acid metabolism. While neurological symptoms are well‐characterized, cutaneous manifestations such as acrodermatitis dysmetabolica (AD) caused by isoleucine deficiency remain underrecognized. This systematic review aimed to summarize reported cases of AD or acrodermatitis enteropathica‐like eruptions in patients with MSUD.

**Methods:**

A systematic search of PubMed, Scopus, and Web of Science was conducted through June 2026. Case reports and studies describing dermatologic findings in MSUD patients with confirmed isoleucine deficiency were included.

**Results:**

19 studies reporting 20 patients were investigated. Most cases involved infants presenting with erythematous, scaly, or erosive lesions primarily in periorificial and acral regions. All patients exhibited low isoleucine levels with normal zinc status. Isoleucine supplementation resulted in clinical improvement in nearly all cases.

**Conclusion:**

Acrodermatitis dysmetabolica is a treatable dermatologic complication of MSUD, typically arising from dietary isoleucine deficiency. Early diagnosis and monitoring of amino acid levels are critical for effective management.

## 1 Introduction

Maple Syrup Urine Disease (MSUD) is a rare autosomal recessive genetic disorder caused by a deficiency in the enzymes responsible for the breakdown of branched‐chain amino acids (BCAAs). The condition is typically diagnosed early in infancy. Management involves lifelong dietary restriction of BCAAs, along with metabolic monitoring to maintain biochemical stability [1]. Due to this enzymatic defect, toxic levels of BCAAs (such as leucine, isoleucine, and valine) and their metabolites accumulate in the blood and tissues, especially in the brain. This accumulation can lead to a wide spectrum of clinical manifestations, ranging from lethargy and poor feeding to skin lesions, seizures, coma, and even death if left untreated [1, 2].

The primary focus has traditionally been on the neurological effects of this metabolic accumulation, with treatment centered on restricting dietary intake of these amino acids [2]. However, dermatological complications such as acrodermatitis dysmetabolica (AD) have emerged as significant concerns during dietary management.

AD is a nutritional deficiency dermatitis that closely resembles Acrodermatitis Enteropathica (AE), a disorder associated with zinc deficiency. Both conditions manifest with similar clinical features, including fatigue, periorificial and acral dermatitis, diarrhea, and alopecia. However, unlike AE, which results from zinc deficiency, AD is primarily caused by deficiencies in essential amino acids, particularly isoleucine [3].

This review aims to consolidate published case reports and studies that examine the association between MSUD and AD, with particular emphasis on clinical manifestations, diagnostic challenges, and therapeutic strategies.

## 2 Methods

Online databases, including Scopus, PubMed, and Web of Science, were searched. This review was conducted up to June 2026. The search strategy was restricted to the following keywords (“Acrodermatitis dysmetabolica” OR “Acrodermatitis enteropathica‐like” OR “Dermatitis” OR “Skin Lesions) AND (“Maple Syrup Urine Disease” OR “MSUD” OR “isoleucine Deficiency”).

After completing the search, the results were imported into EndNote reference management software. In the first step, duplicated records were removed. Two reviewers independently screened the titles and abstracts after duplicate removal. Potentially eligible full texts were then assessed independently by the same reviewers. Disagreements were resolved through discussion and, when necessary, consultation with a third reviewer. Titles were reviewed, and irrelevant ones were excluded. Subsequently, abstracts were assessed, and studies with irrelevant content were also excluded. Efforts were made to obtain the full texts of potentially relevant articles, using institutional access or by contacting the corresponding authors. Finally, all included full‐text articles were reviewed in detail for comprehensive data extraction and summarization.

We included case reports, case series, and observational studies that reported patients with MSUD presenting with dermatological manifestations, specifically AD or AE‐like skin lesions. Studies were required to provide clinical, laboratory, or histopathological confirmation of isoleucine deficiency.

**Figure 1:**
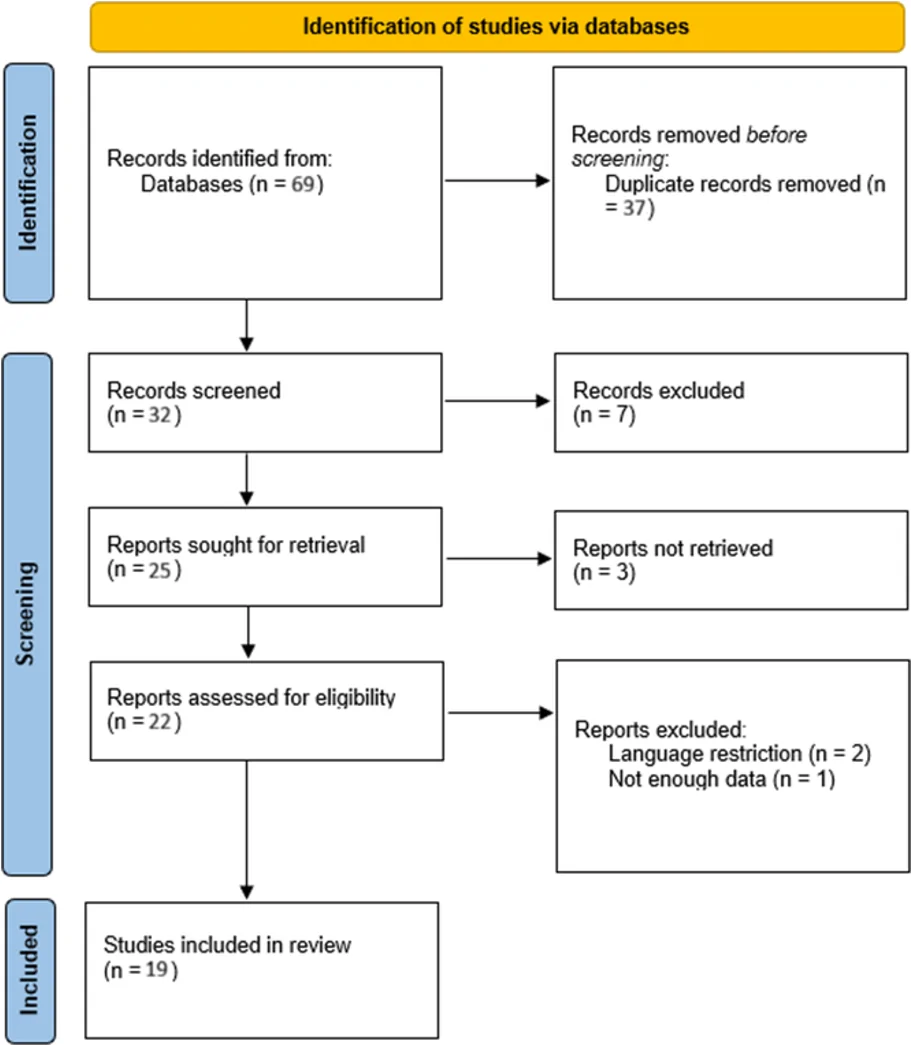
PRISMA flow diagram showing identification, duplicate removal, title/abstract screening, full‐text eligibility assessment, and final inclusion of studies reporting acrodermatitis dysmetabolica eruptions in patients with maple syrup urine disease.

We excluded studies that did not involve MSUD patients, those without relevant dermatological findings, review articles, editorials, conference abstracts without sufficient patient data, animal studies, and articles with incomplete clinical data. The flowchart of the included articles can be seen in Figure 1.

### 2.1 Quality Assessment

The quality of the included studies was assessed using the Joanna Briggs Institute (JBI) critical appraisal checklists, tailored to each study design. Each checklist consists of a series of questions evaluating key methodological aspects such as selection bias, confounding factors, and data analysis. Items were scored as “Yes,” “No,” “Unclear,” or “Not applicable.”

A total quality score was calculated based on the number of “Yes” responses. Studies were then categorized according to their overall methodological quality as high, moderate, or low quality based on predefined cut‐off points. This systematic assessment ensured a rigorous evaluation of the risk of bias and the reliability of study findings. Quality assessment is presented in Table 1.

**Table 1:** Quality assessment of the included studies.

Study	1. Demographics	2. History Timeline	3. Clinical Condition	4. Diagnostic Tests	5. Intervention Described	6. Outcome Described	7. Adverse Events Reported	8. Lessons Provided	Overall Quality
Sanjurjo et al., 1986 [4]	Yes	No	Yes	Yes	Yes	Yes	No	Yes	Moderate
Alkhayal et al., 2023 [5]	Yes	Yes	Yes	Yes	Yes	Yes	No	Yes	High
El‐Darouti et al., 1994 [6]	Yes	No	Yes	Yes	Yes	Yes	No	Yes	Moderate
Flores et al., 2015 [3]	Yes	Yes	Yes	Yes	Yes	Yes	No	Yes	High
Campuzano‐Garcia et al., 2019 [7]	Yes	Yes	Yes	Yes	Yes	Yes	No	Yes	High
Giacoia et al., 1993 [8]	Yes	Yes	Yes	Yes	Yes	Yes	No	Yes	High
Gupta et al., 2014 [9]	Yes	No	Yes	No	Yes	Yes	Yes	Yes	Moderate
Hou et al., 2013 [10]	Yes	Yes	Yes	Yes	Yes	Yes	No	Yes	High
Kazandjieva et al., 2015 [11]	Yes	Yes	Yes	Yes	Yes	Yes	No	Yes	High
Koch et al., 1993 [12]	Yes	Yes	Yes	Yes	Yes	Yes	No	Yes	High
Northup et al., 1993 [13]	Yes	Yes	Yes	Yes	Yes	Yes	No	Yes	Moderate
Ozturk et al., 2008 [14]	Yes	Yes	Yes	Yes	Yes	Yes	No	Yes	High
Puzenat et al., 2004 [15]	Yes	Yes	Yes	Yes	Yes	Yes	No	Yes	High
Rivera‐Comparán et al., 2023 [16]	Yes	No	Yes	No	Yes	Yes	No	Yes	Moderate
Ross et al., 2015 [17]	Yes	Yes	Yes	Yes	Yes	Yes	No	Yes	High
Santaliz‐Ruiz et al., 2024 [14]	Yes	Yes	Yes	Yes	Yes	Yes	No	Yes	High
Templier et al., 2006 [18]	Yes	Yes	Yes	Yes	Yes	Yes	No	Yes	High
Uaariyapanichkul et al., 2017 [19]	Yes	Yes	Yes	Yes	No	No	No	Yes	High
Kahraman et al., 2025 [20]	Yes	Yes	Yes	Yes	Yes	Yes	No	Yes	High

## 3 Results

This review included published case reports from 1986 to 2026 originating from various countries around the world. After our primary search, 69 studies were identified that complied with our keywords. 32 studies remained after removing duplicates, and after a thorough screening, 19 studies were retrieved and included in this review.

Most patients were under 1 year of age, with only one case involving an adult—a 24‐year‐old woman. Both sexes were represented among the reported cases. The mean age at presentation was approximately 21.8 months. Of the 20 patients, 9 were female, accounting for 45% of the total.

The included studies and their characteristics are presented in Table 2.

**Table 2:** Details and characteristics of the included studies.

Authors, Year	Country	Age at presentation/Sex	Clinical Manifestations	Paraclinical Investigations	Treatment and management
Sanjurjo et al., 1983 [4]	Spain	Neonate/F	Cutaneous lesions in the extremities and diaper area	Low isoleucine levels and high levels of leucine	Dietary recommendations and supplementation
Alkhayal et al., 2023 [5]	Saudi Arabia	2 months/M	Multifocal seizures/scaly erythematous plaques over perioral area, napkin area, bilateral proximal upper and lower extremities, acral hands/hair loss	normal zinc level and valine, low isoleucine and elevated leucine/ Histological examination of thigh skin punch biopsy showed parakeratosis with underlying pale keratinocytes in the upper epidermis, spongiosis and psoriasiform hyperplasia	isoleucine supplementation and leucine restriction, and topical Mometasone 0.1% ointment twice daily for 14 days./the lesions were completely resolved after 4 weeks.
El‐Darouti et al., 1994 [6]	Egypt	6 months/F	Lethargy/failure to thrive/Acral erythema and exfoliative dermatitis of the perioral and anogenital regions/sweet odor of the urine	Normal zinc and biotin levels/low isoleucine levels	Isoleucine supplementation resulted in the quick resolution of the lesions
Flores et al., 2015 [3]	USA	3 months/M	Poor feeding/diarrhea/hair loss/skin lesions	Normal zinc and valine levels/low isoleucine, and high leucine levels	The isoleucine supplementation was increased from 50 to 300 mg daily and resulted in the resolution of lesions.
Campuzano‐Garcia et al., 2019 [7]	Mexico	1 year old/F	erythematous macules and papules with a predominance in the diaper area, flexural sites, and proximal thighs/hypotonia	Normal zinc and ammonia/low leucine levels	Leucine supplementation resulted in the resolution of the lesions
Giacoia et al., 1993 [8]	USA	1 months old/M	Macular and vesicular lesions on the extremities and trunk	Normal zinc and valine levels/low isoleucine, and high leucine levels	Supplementation with 85 mg/kg per day of both isoleucine and valine, and a resolution of lesions within 7 days
Gupta et al., 2014 [9]	India	49 days‐old boy/M	erythematous scaly lesions involving the periorofacial and perianal regions and extremities	—	Isoleucine supplementation
		21 days‐old boy/M	Seizures/hypotonia/acrodermatitis enteropathica‐like lesions	—	The lesions were unresponsive to zinc supplantation/. Unfortunately, the baby expired due to sepsis
Hou et al., 2013 [10]	Taiwan	2 months‐old/male	Anemia/diarrhea/dermatitis in limbs, trunk, and peritoneum	Low Valine and isoleucine/slightly low levels of zinc	Subsequent supplementation of BCAAs (115 mg/kg/day of leucine, 90 mg/kg/day of isoleucine, and 100 mg/kg/day of valine, combined with other amino acids and rapid resolution of lesions
Kazandjieva et al., 2015 [11]	Bulgaria	4 months‐old/F	Diarrhea/hypotension/maculopapular exanthema on the scalp, trunk, upper and lower extremities, and exfoliative dermatitis of the perioral and anogenital regions	normal zinc serum level with deficiencies of leucine and valine	BCAA supplementation and skin hygiene
Koch et al., 1993 [12]	USA	9 days‐old/F	Neurologic symptoms/scaly erythematous dermatitis	A skin biopsy specimen showed a superficial, perivascular lymphohistiocytic infiltrate with erosion of the outer epidermis/low isoleucine and low valine levels, and high leucine levels	Supplementation with isoleucine, 35 mg/kg/day, and valine, 40 mg/kg/day, was begun. During the succeeding days, the rash became less erythematous and began to desquamate.
		2 weeks‐old/F	diaper dermatitis and an erythematous scaling eruption/alopecia	Normal zinc levels/dramatically low isoleucine level, elevated leucine level, and a mildly elevated valine level	Isoleucine supplementation and resolution of the lesions
Northup et al., 1993 [13]	USA	4.5 months old/M	Growth retardation/mild hepatomegaly/skin lesions	Elevated valine and leucine levels and low isoleucine levels	—
Ozturk et al., 2008 [14]	Turkey	4 years‐old/M	Diarrhea/alopecia/moist, erythematous lesions on his face, back, and extremities	Normal zinc, valine, and leucine, with low levels of isoleucine	A high‐calorie diet with isoleucine led to a prompt improvement of the boy's skin lesions
Puzenat et al., 2004 [15]	France	28 days‐old/M	Diarrhea/significant pallor/exfoliative dermatitis (perioral, anogenital, and neck regions)/stomatitis	Normal zinc levels, elevated leucine and valine levels, and a low level of isoleucine	Addition of low‐dose isoleucine to the diet and Rapid resolution of symptoms.
Rivera‐Comparán et al., 2023 [16]	Mexico	5 months‐old/M	lethargy/disseminated erythematosquamous plaques/alopecia	—	Isoleucine supplementation. Following this adjustment, the patient's skin lesions improved within seven days.
Ross et al., 2015 [17]	India	12 days‐old/M	excoriative skin lesions in the perianal and perioral regions, and both feet	Normal zinc level/elevated leucine and valine/low isoleucine levels	Modified diet and the resolution of the lesions
Santaliz Ruiz et al., 2024 [14]	Puerto rico	24 years‐old/F	nonpruritic cutaneous eruption on neck, anogenital region, face, and extremities/diarrhea, and hair loss for the past 2 weeks	Low levels of leucine and valine/normal isoleucine/hypoalbuminemia/low levels of zinc	The patient received zinc, leucine, and valine supplementation.
Templier et al., 2006 [18]	France	5 months‐old/F	periorificial and acral dermatitis, diarrhea, erythematous and erosive plaques in axillary, inguinal, and perineal regions, acral erythema	High leucine levels and low valine and isoleucine/normal zinc levels/Skin biopsy showed epidermal thickening with dystrophic, clarified keratinocytes and parakeratosis	Reintroduction of valine and isoleucine at 150 mg/day, leading to normalization of amino acid levels and rapid resolution of skin lesions
Uaariyapanichkul et al., 2017 [19]	Thailand	45 days‐old/M	erythematous macules and papules on the forehead, hemorrhagic crusts at both upper and lower lips, as well as well‐defined erythematous patches at both cheeks, the flexor part of the neck, forearms, trunk, and the anogenital area	Normal zinc and valine values, low isoleucine, and high levels of leucine	isoleucine supplementation of 100 mg/day. The patient had been on continuous venous hemofiltration for a 48‐h period to reduce elevated leucine levels and rapid improvements.
Kahraman et al., 2025 [20]	Turkey	30 months old/F	periorificial and acral dermatitis, erythematous and erosive plaques	Severe isoleucine deficiency secondary to a protein‐restricted diet	Dietary adjustment with improvement of AD lesions

The clinical presentations of most patients were similar. They exhibited erythematous, scaly, or erosive skin lesions in the periorificial regions (perioral and perianal), extremities, diaper area, and flexural folds. Additional symptoms included alopecia, diarrhea, and irritability. In some cases, neurological features such as hypotonia or seizures were also presented [5, 9, 12]. These cutaneous manifestations typically emerged during or after the initiation of dietary treatment for MSUD, particularly when BCAA levels were not carefully monitored.

Laboratory findings commonly revealed low plasma isoleucine levels, often accompanied by elevated leucine levels. Zinc levels were normal in nearly all cases, thereby ruling out AE and pointing toward some other underlying cause, which turned out to be amino acid deficiency. Histopathological investigations in the reported cases predominantly demonstrated parakeratosis, psoriasiform hyperplasia, and pale keratinocytes [5, 12, 18]. Valine levels were variably reported across studies, ranging from low to normal and, in some cases, elevated. This variability highlights the need to interpret valine together with leucine, isoleucine, clinical findings, and dietary history.

Most cases showed favorable outcomes following correction of amino acid imbalances. Supplementation with the deficient amino acids typically led to resolution of the lesions within a few weeks. In several cases, topical corticosteroids were also administered to provide symptomatic relief. However, one infant was eventually diagnosed with sepsis and died, highlighting the potential consequences of delayed diagnosis and the severity of metabolic instability in neonates with MSUD [9].

## 4 Discussion

In this review, we assessed 19 case reports that described the correlation between MSUD and dermatological manifestations, particularly AD. Differentiating between AE and AD is clinically challenging due to their similar presentations [6, 14, 15]. Misdiagnosis can result in inappropriate treatment, as zinc supplementation does not resolve skin lesions caused by amino acid deficiencies. Therefore, in MSUD patients presenting with dermatitis, it is essential to assess plasma amino acid levels, particularly to detect isoleucine deficiency.

However, in MSUD, AD is primarily attributed to isoleucine deficiency induced by the dietary restrictions necessary for managing the disorder. The most prominent mechanism involved was suggested to be impaired keratinocyte growth and protein synthesis.

Isoleucine is essential for the normal growth and differentiation of keratinocytes. When plasma isoleucine is low, protein synthesis in these skin cells is disrupted, leading to characteristic dermatitis [21]. On the other hand, histopathology often shows psoriasiform hyperplasia, parakeratosis, vacuolar changes, and epidermal pallor, indicative not only of halted protein synthesis but also of impaired barrier repair, spongiosis, and cell cohesion [22].

Several case reports have noted that infants developed characteristic skin lesions during periods of specialized formula intake. In these cases, plasma amino acid analysis consistently revealed low isoleucine levels with normal zinc concentrations [15, 17, 18, 19].

In patients with MSUD, AD should be considered a clinical warning sign of unbalanced dietary management and/or inadequate metabolic follow‐up. This imbalance may occur because of limited access to serial plasma amino acid testing, irregular follow‐up visits, caregiver‐related or socioeconomic barriers, and limited availability of specialized centers for inborn errors of metabolism. Therefore, the appearance of AD should prompt clinicians not only to treat the skin eruption but also to reassess dietary intake, adherence, growth, access to metabolic monitoring, and the frequency of specialist follow‐up [23, 24].

Clinicians should actively look for earlier signs of nutritional imbalance before overt AD develops, including poor weight gain for age, stunted growth, generalized candidiasis, persistent napkin dermatitis, diarrhea, alopecia, irritability, and poor feeding. Importantly, AD in MSUD may occur in the setting of elevated leucine together with low isoleucine and/or valine. Therefore, high leucine should not automatically lead to further leucine restriction without evaluating the complete plasma amino acid profile. Management should focus on restoring amino acid balance, particularly by supplementing deficient isoleucine and, when indicated, valine, while maintaining safe leucine control under specialist metabolic supervision [25, 26]. Caregiver education and reassurance are essential because early reporting of subtle signs of malnutrition should be encouraged and should not be interpreted as caregiver failure; rather, it allows timely dietary adjustment before severe dermatitis, infection, or metabolic instability develops.

The primary intervention for AD in patients with MSUD involves careful dietary management to ensure sufficient isoleucine intake while maintaining overall metabolic stability and preventing MSUD complications. Supplementation with isoleucine, and in some instances valine, has been shown to effectively resolve skin lesions associated with AD. In some cases, additional interventions such as parenteral nutrition may be required, particularly when oral supplementation is inadequate due to malabsorption, intercurrent illness, or during metabolic decompensation [19].

Dietary management of MSUD aims to maintain leucine within a safe therapeutic range while preventing deficiency of other essential BCAAs. Published nutrition management guidance emphasizes dietary leucine restriction, BCAA‐free medical foods, targeted supplementation with isoleucine and valine, and frequent clinical and biochemical monitoring. In patients who develop AD, treatment decisions should be guided by absolute plasma amino acid levels, trends over time, dietary intake, growth parameters, and clinical findings. Leucine: alanine and leucine: phenylalanine ratios may help identify or interpret MSUD‐related metabolic imbalance, particularly in screening or diagnostic contexts; however, AD‐specific ratio cutoffs have not been established. Therefore, these ratios should not replace direct assessment of leucine, isoleucine, and valine levels when evaluating suspected AD in MSUD [23, 24, 25, 27].

Beyond MSUD, AD has been reported in various metabolic disorders. Case reports have described AE‐like eruptions secondary to metabolic disorders, including methylmalonic acidemia, phenylketonuria, ornithine transcarbamylase deficiency, glutaric acidemia, citrullinemia, and propionic acidemia.

These studies found that these patients also exhibited skin lesions similar to those seen in zinc deficiency, but with normal zinc levels, pointing to a metabolic rather than a micronutrient etiology. Notably, all reported cases showed marked clinical improvement following dietary modifications specific to their underlying metabolic disorders [28, 29, 30]. These findings underscore the importance of considering amino acid and nutrient imbalances as potential contributors to dermatologic symptoms in metabolic diseases, and highlight the need for individualized nutritional interventions.

These comparative studies highlight the importance for clinicians to recognize AD in patients with various inborn metabolic disorders presenting with AE‐like skin lesions. Accurate diagnosis requires a comprehensive evaluation of the patient's metabolic and nutritional status, including metabolite blood levels, and a detailed dietary history. This dermatological manifestation may reflect an iatrogenic abnormal metabolic status and should be noted. precise identification, followed by targeted nutritional interventions, can lead to rapid and effective resolution of the skin lesions and prevent further complications.

## 5 Conclusion

The occurrence of AD in patients with MSUD highlights the delicate balance required in dietary management to prevent both metabolic symptoms and nutritional deficiencies. Regular assessment of plasma amino acid levels, particularly isoleucine, is essential for individuals with MSUD to effectively prevent and manage dermatological manifestations. Further research into the pathophysiology of AD in metabolic disorders may provide better insights for improved prevention and treatment strategies, ultimately enhancing patient outcomes.

## Author Contributions

**Bahareh Abtahi‐naeini:** conceptualization, supervision, writing – review and editing, writing – original draft. **Motahar Heidari‐Beni:** investigation, writing – original draft, writing – review and editing, validation. **Noushin Rostampour:** conceptualization, methodology, investigation, writing – review and editing. **Naeimeh Davoudi:** writing – original draft, writing – review and editing, methodology, investigation. **Sarah Seyedyousefi:** conceptualization, investigation, methodology, writing – review and editing, writing – original draft.

## Funding

The authors have nothing to report.

## Ethics Statement

The authors have nothing to report.

## Consent

The authors have nothing to report.

## Conflicts of Interest

The authors declare no conflicts of interest.

## Transparency Statement

Bahareh Abtahi‐naeini affirms that this manuscript is an honest, accurate, and transparent account of the study being reported; that no important aspects of the study have been omitted; and that any discrepancies from the study as planned have been explained.

## Data Availability

The data that support the findings of this study are available from the corresponding author upon reasonable request.
